# Neuromuscular Junction Damage in the Calf Muscles of Patients With Advanced Peripheral Artery Disease

**DOI:** 10.1111/nan.70008

**Published:** 2025-02-24

**Authors:** Huiyin Tu, Ali H. Hakim, Julian K. Kim, Zhen Zhu, Yuqian Tian, Iraklis I. Pipinos, Yu‐Long Li

**Affiliations:** ^1^ Department of Emergency Medicine University of Nebraska Medical Center Omaha Nebraska USA; ^2^ Department of Surgery University of Nebraska Medical Center Omaha Nebraska USA; ^3^ Department of Surgery and VA Research Service VA Nebraska‐Western Iowa Health Care System Omaha Nebraska USA; ^4^ Department of Cellular and Integrative Physiology, University of Nebraska Medical Center Omaha Nebraska USA

**Keywords:** lower limb, morphology, neuromuscular junction, patients, peripheral artery disease

## Abstract

**Aims:**

Peripheral artery disease (PAD) reduces blood flow to the legs and causes severe muscle and leg dysfunction for PAD patients. Skeletal muscle contractile function is dependent on the health of the muscle itself and that of the neuromuscular junction (NMJ) on the muscle membrane.

**Methods:**

To determine whether the NMJ, including the motor nerve terminals and nicotinic acetylcholine receptors (nAChR), is damaged in PAD, gastrocnemius muscles were collected from 3 controls and 13 PAD patients to capture images from 331 control NMJs and 512 PAD NMJs.

**Results:**

For the motor nerve terminals, there were more denervated nAChR clusters and fewer nerve terminal occupancies in NMJs in PAD patients, compared with controls. For the nAChR clusters in the NMJs, the area per nAChR cluster was 369.3 ± 6.7 versus 225.2 ± 5.3 μm^2^, the area per fragment was 195.9 ± 9.2 versus 107.1 ± 3.1 μm^2^, the number of fragments per nAChR cluster was 2.3 ± 0.1 versus 3.2 ± 0.1, the nAChR cluster area per endplate area was 75.7 ± 1.6 versus 55.7 ± 1.1%, total distance of fragments per nAChR cluster was 4.6 ± 0.4 versus 8.8 ± 0.8 μm, and the fragmented nAChR clusters were 7.6% versus 21.6% of total nAChR clusters in controls versus PAD patients, respectively (*p* < 0.05 in all parameters).

**Conclusions:**

Our data demonstrate deterioration of the motor nerve terminals and nAChR clusters, which may compromise neuromuscular transmission, and contribute to the severe leg dysfunction observed in patients with PAD.

## Graphical abstract

In severe peripheral artery disease (PAD), the neuromuscular junction (NMJ) is damaged, with deterioration of motor nerve terminals and fragmentation of the nicotinic acetylcholine receptor clusters. This likely contributes to the severe leg dysfunction of patients with advanced PAD. Targeting damaged NMJs could offer a novel approach to improving skeletal muscle and leg function in PAD patients.

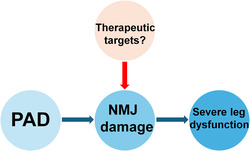

**Summary**

- Neuromuscular junction damage in patients with advanced peripheral artery disease includes deterioration of the motor nerve terminals and fragmentation of the nicotinic acetylcholine receptor clusters.
- Neuromuscular junction damage is involved in the severe leg dysfunction in patients with advanced peripheral artery disease.

## 1 Introduction

Peripheral artery disease (PAD) is a manifestation of systemic atherosclerosis characterised by progressive narrowing and occlusion of the arteries perfusing the lower extremities [1]. Over 200 million people worldwide suffer from PAD with an annual mortality rate of 4%–6% [2, 3, 4]. Based on the Fontaine classification, asymptomatic patients comprise Stage I of the disease while symptomatic PAD patients present with claudication (Stage II PAD, debilitating muscle discomfort with walking), ischaemic rest pain (Stage III PAD, severe limb pain at rest) and tissue loss (Stage IV PAD, non‐healing ulcers and gangrene). Frequently, patients with Stage III and Stage IV PAD are grouped together in a category reported as chronic limb threatening ischaemia (CLTI) [5, 6]. Current standard therapies, including pharmacological interventions, supervised exercise therapy programmes, operative revascularisation and amputation, offer modest benefits for PAD patients and have significant limitations [5, 6, 7]. Consequently, there is an urgent need for new advances and therapies to enhance limb function and healing in PAD patients, ultimately improving their quality of life.

The most common manifestation of PAD in all affected patients, including those who are asymptomatic, is a significant impairment in the function of the lower extremity and its skeletal muscles [8, 9, 10, 11]. Skeletal muscle contractile function is dependent not only on the health of the muscle tissue itself but also on the integrity of the neuromuscular junction (NMJ), which facilitates motor nerve‐muscle transmission [12, 13, 14]. To date, most research on the pathophysiology of PAD has focused on abnormalities of the blood vessels and skeletal muscles of the affected lower extremities, while the effects of PAD on the NMJ have remained largely unexplored. The structure of the NMJ consists of intact nicotinic acetylcholine receptor (nAChR) clusters, and motor nerve terminals that innervate these clusters, forming synapses essential for motor nerve‐muscle signal transmission [15]. Motor nerve denervation and NMJ dysfunction, such as fragmentation of nAChR clusters, are hallmarks of various neuromuscular diseases [16, 17, 18, 19, 20], including NMJ damage from acute and chronic ischaemia‐reperfusion injury [21, 22, 23]. The potential involvement of NMJ injury in PAD may represent a significant contributor to the pathophysiology of the disease and could serve as a novel target for developing effective treatments for PAD patients. The present study investigated the hypothesis that significant NMJ damage occurs in the calf muscles of patients with advanced PAD presenting with CLTI.

## 2 Materials and Methods

### 2.1 Patients and Amputation Specimens

The study was approved by the Institutional Review Boards at the Nebraska‐Western Iowa Veteran Affairs Health Care System and the University of Nebraska Medical Center. Thirteen patients with PAD diagnosed with CLTI were recruited through the vascular surgery clinics of these institutions. The cohort comprised 10 males (76.9%), with a mean age of 66.3 ± 15.2 years, height of 173.5 ± 9.3 cm, and body mass index of 27.1 ± 8.1 (Table 1). For every patient, the diagnosis of PAD was based on a comprehensive evaluation including medical history, physical examination, measurement of lower limb haemodynamics and computerised or standard arteriography demonstrating significant stenoses and/or occlusions in the arteries supplying the lower extremities. The Fontaine classification system was used to categorise PAD patients based on their clinical presentation. One Stage III PAD patient presented with ischaemic rest pain in the foot, whereas 12 Stage IV PAD patients presented with ischaemic ulceration or gangrene of the affected limb. All PAD patients underwent below‐knee amputation surgery for the treatment of their disease, and gastrocnemius muscle samples were obtained from all patients at the time of this operation. Three control subjects were also recruited. They were all male with a mean age of 45.7 ± 19.1 years, height of 178.6 ± 5.9 cm and body mass index of 28.3 ± 6.1 (Table 1). The control patients were carefully selected to avoid any underlying lower extremity pathology that potentially has a neuropathological component. One muscle sample was obtained from a patient who underwent below‐knee amputation for osteomyelitis of a non–healing heel ulcer obtained from previous trauma to the foot; another muscle sample was obtained from a patient who underwent left superficial femoral artery to below‐knee popliteal bypass surgery for a popliteal aneurysm, and the last muscle sample was obtained at the time of organ tissue donation from a trauma patient with no medical history who suffered a severe head injury from motor vehicle collision. All human subjects or a designated power of attorney provided written, informed consent for tissue donation. To achieve robust and unbiased data, all human samples were blinded during experimentation and data acquisition until statistical analysis. Additionally, two investigators independently assessed the NMJs in all samples.

**TABLE 1:** Demographics and disease characteristics of the PAD and control groups.

	PAD stage III/IV (*N* = 13)	Control (*N* = 3)	*p*
Age, mean [SD]	66.3 [15.2]	45.7 [19.1]	0.030
Sex, no. [%]			
Male	10 [76.9]	3 [100]	0.511
Female	3 [23.1]	0 [0]	0.511
Race, no. [%]			
White	9 [69.2]	2 [66.7]	0.705
African American	2 [15.4]	1 [33.3]	0.489
Asian or Pacific Islander	2 [15.4]	0 [0]	0.650
Height (cm), mean [SD]	173.5 [9.3]	178.6 [5.9]	0.193
Body mass index, mean [SD]	27.1 [8.2]	28.3 [6.1]	0.410
Co‐morbidities, no. [%]			
Hypertension	10 [76.9]	1 [33.3]	0.214
Diabetes	10 [76.9]	0 [0]	0.036
Dyslipidaemia	6 [46.2]	0 [0]	0.214
Coronary artery disease	4 [30.8]	0 [0]	0.393
Former smoker	6 [46.2]	0 [0]	0.214
Current smoker	6 [46.2]	2 [66.7]	0.500
Ankle‐brachial index, mean [SD]	0.4 [0.2]	1.1 [0.1]	<0.001
Transcutaneous oxygen pressure (mmHg), mean [SD]			
At calf level	39.4 [8.7]		
At foot level	24.6 [18.8]		
Arterial occlusion level			
Combined femoropopliteal and tibial occlusive disease	8 [61.5]		
Tibial occlusive disease	5 [38.5]		
PAD stage			
Stage III	1 [7.7]		
Stage IV	12 [92.3]		

### 2.2 Immunohistochemistry of the NMJ

We used immunofluorescence labelling to analyse NMJ morphology, following protocols previously established in our laboratory [21, 22, 24]. Human gastrocnemius samples were rapidly isolated and post‐fixed with 4% paraformaldehyde for 15 min, followed by incubation with 0.1 M glycine for 15 min. To facilitate probe penetration, each muscle sample was divided into 8–10 small longitudinal segments and then permeabilised in −20°C methanol for 10 min. After blocking with PBS containing 0.5% Triton (BP151, Thermo Fisher Scientific, Waltham, MA) and 1% BSA (A7888, Sigma, St. Louis, MO) for 1 h, the segments of the gastrocnemius muscle were incubated overnight at 4°C in a cocktail of primary antibodies. These included mouse anti‐neurofilament 200 (NF‐200, N0142, Sigma‐Aldrich, St. Louis, MO) and rabbit anti‐synaptophysin (MA5–16402, Thermo Fisher Scientific, Waltham, MA) antibodies, for axon and nerve terminal labelling, respectively. Subsequently, the muscle segments were incubated overnight at 4°C with Alexa Fluor 594 labelled donkey anti‐mouse (A21203, Thermo Fisher Scientific, Waltham, MA) and anti‐rabbit (A21207, Thermo Fisher Scientific, Waltham, MA) IgGs, along with Alexa Fluor 488 labelled α‐bungarotoxin (α‐BTX, B13422, Thermo Fisher Scientific, Waltham, MA) for nAChR labelling.

Images of muscle segments mounted on glass slides were captured using a laser scanning confocal microscope (Zeiss LSM 800) to analyse immunohistochemically labelled NMJs, including motor nerve terminals and nAChR clusters. In each muscle segment, five different regions were selected to obtain Z‐stack images of the NMJ. All analyses were done on *en‐face* NMJs, confirmed by 3D reconstruction using Zen (blue edition). Z‐stack images of the NMJs were processed in Zen (black edition) to create 2D reconstructions, yielding maximum intensity projections that represent the maximum projected area of the NMJs. Finally, NMJ images were analysed and quantified using ImageJ software (NIH Image).

In the NMJ, the percentage of motor nerve innervation and motor nerve occupancy was quantified by measurements of nerve terminals labelled with NF‐200 and synaptophysin and nAChRs labelled with α‐BTX. Motor nerve occupancy was used to describe the overlap between presynaptic nerve terminals and postsynaptic nAChR clusters and calculated as a percentage of presynaptic nerve terminal area versus postsynaptic nAChR cluster area. The endplate with or without labelling of neurofilament and synaptophysin was defined as an innervated or denervated endplate, respectively. The nAChR areas in NMJs labelled with α‐BTX were used to calculate the whole area per nAChR cluster, the number of discrete fragments per nAChR cluster, the area per fragment, the nAChR area per endplate area, the total distance of fragment, and the percentage of fragmented nAChR clusters in total nAChR clusters. A fragmented nAChR cluster was defined when the number of discrete fragments per nAChR cluster ≥ 5. Endplate area was calculated as the maximum projected area of the nAChR cluster. The total distance of nAChR fragments was the sum of the minimum distance of one fragment to the next.

### 2.3 Statistical Analysis

Data are presented as means ± SEM. SigmaStat 12 was used for statistical analyses. Unpaired *t*‐tests and Fischer's exact tests were used to determine statistical significance for two‐group comparisons. Normal distribution of data was confirmed using the Kolmogorov–Smirnov test, and equal variance was assessed with Levene's test. Statistical significance was accepted when *p* < 0.05.

## 3 Results

### 3.1 Morphology of the NMJ in Control Subjects

We examined a total of 331 NMJs from gastrocnemius muscle samples obtained from three control subjects. The analysis of these NMJs provided a baseline for comparison with PAD patients presenting with CLTI.

**FIGURE 1:**
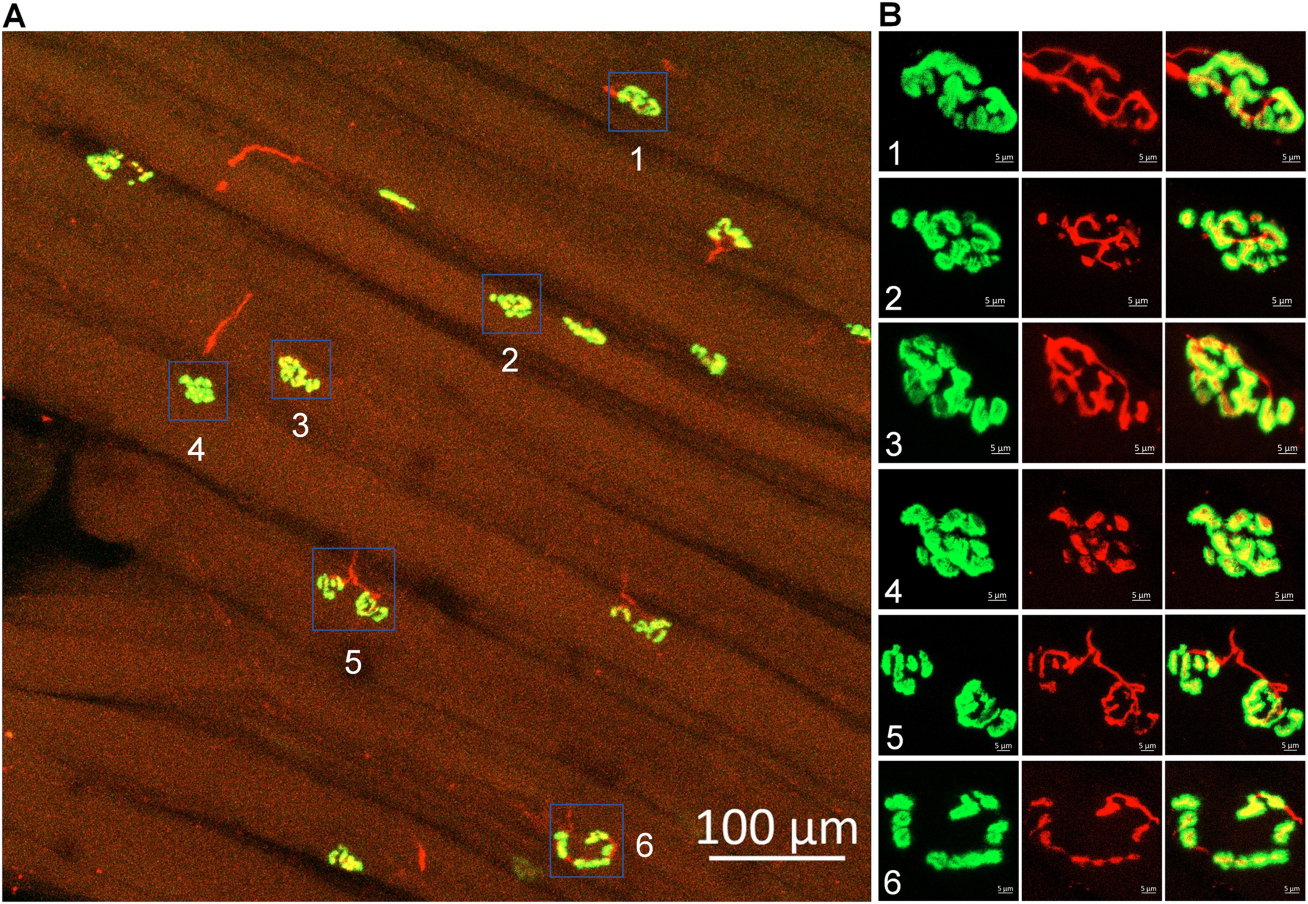
Representative morphology of NMJs in the gastrocnemius of a trauma patient. The insets in the right panel (B) provide magnified views of the NMJs seen in the left panel (A). Motor nerve axons and terminals (red colour) were labelled using antibodies against neurofilament 200 and synaptophysin. Nicotinic acetylcholine receptors (green colour) were labelled using Fluor 488‐α‐bungarotoxin. In the normal NMJs, the nAChR clusters (green colour) exhibit an intact, pretzel‐like structure, comprised two to three interconnected fragments. The motor nerve terminals (red colour) demonstrate precise overlap with a nAChR cluster (green colour) to form synapses for signal transmission.

In the young trauma donor (Figure 1), we analysed 105 NMJs. The two primary components of the NMJs, including motor nerve terminals and the nAChR clusters, were labelled with neurofilament 200/synaptophysin and α‐BTX, respectively (Figure 1). The nAChR clusters predominantly exhibited an intact, pretzel‐like structure, typically comprising two to three interconnected fragments (Figure 1). Motor nerve terminals demonstrated precise overlap with nAChR clusters, indicating proper innervation (Figure 1).

**FIGURE 2:**
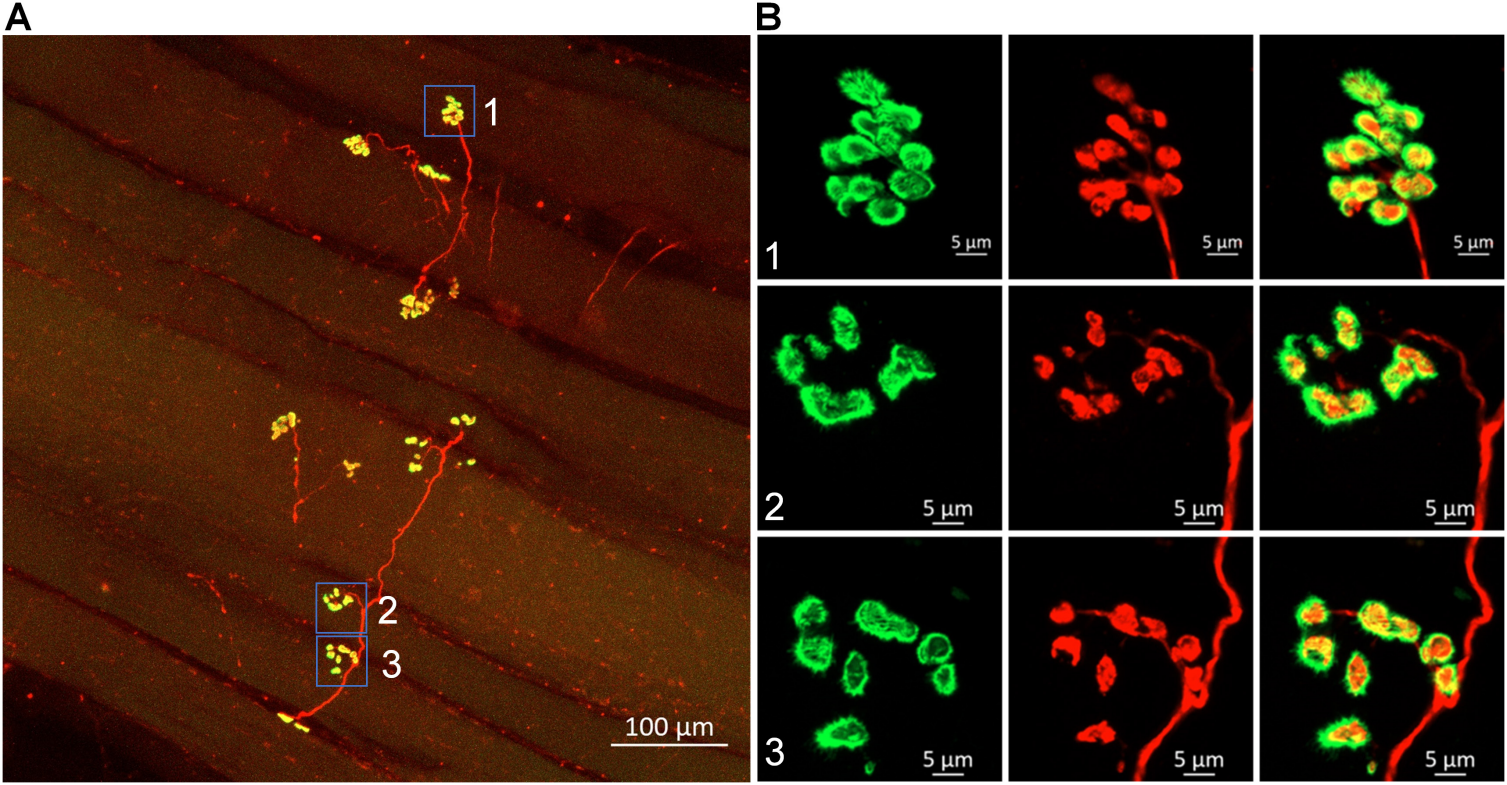
Representative morphology of NMJs in the gastrocnemius of a control patient who underwent a vascular bypass operation due to a popliteal aneurysm. The insets in the right panel (B) provide magnified views of the NMJs seen in the left panel (A). While most nAChR clusters in this older control patient remained intact, pretzel‐like structure (B, Panel 1), a small proportion displayed a more dispersed structure (B, Panels 2 and 3). Importantly, nearly all nAChR clusters were innervated by motor nerve terminal boutons (A).

From the two older control subjects, we examined an additional 226 NMJs (Figure 2). While most nAChR clusters in these samples remained intact (Figure 2B, Panel 1), a small proportion displayed a more dispersed structure (Figure 2B, Panels 2 and 3). Importantly, nearly all nAChR clusters were innervated by motor nerve terminal boutons (Figure 2A). The 331 NMJs obtained from the three control patients were used as a basis for mean morphology that was subsequently compared with PAD patients with CLTI (Figure 5).

### 3.2 Alterations in the Morphology of the NMJ in PAD Patients With CLTI

**FIGURE 3:**
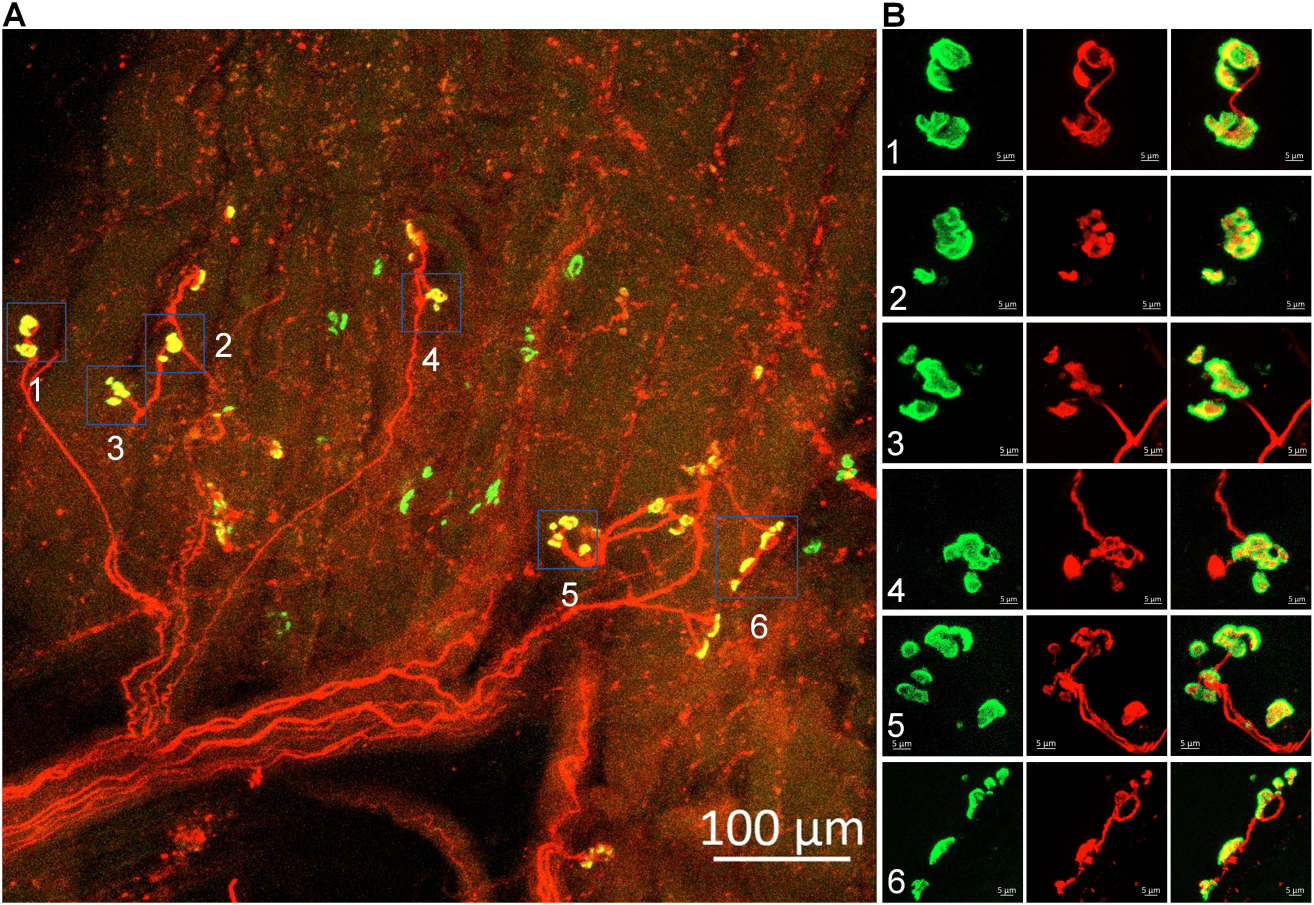
Representative morphology of the NMJs in the gastrocnemius from a Stage IV patient with PAD who underwent a below‐knee amputation. The insets in the right panel (B) provide magnified views of the NMJs seen in the left panel (A). The majority of NMJs in this patient exhibit significant structural alterations. These changes are characterised by fragmented nAChR clusters, with only a few dispersed fragments of nAChRs forming each cluster (green colour, B). Notably, some nAChR clusters had lost the motor nerve terminal innervation (red colour, B).

**FIGURE 4:**
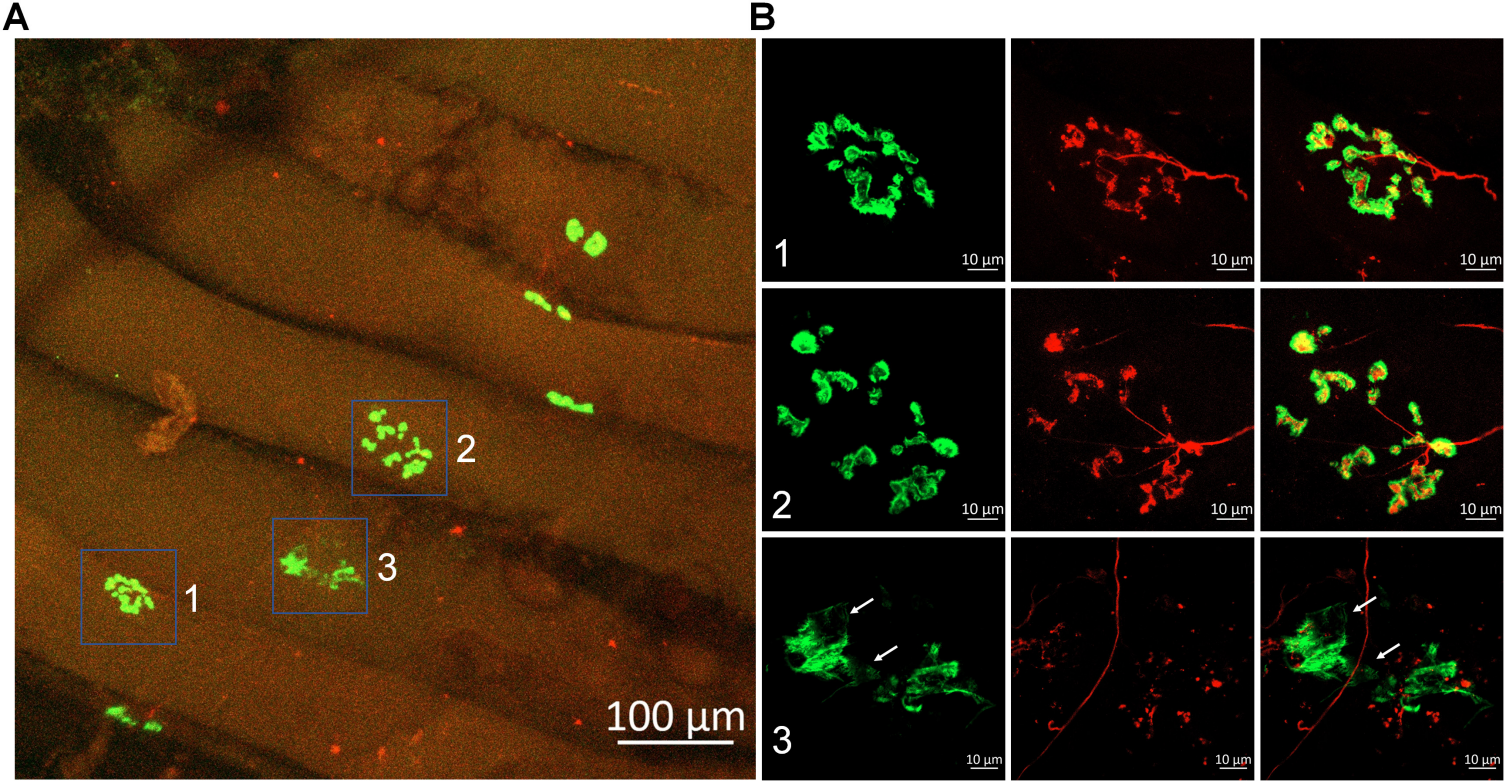
Representative morphology of NMJs in the gastrocnemius from another Stage IV patient with PAD who underwent a below‐knee amputation. The insets in the right panel (B) provide magnified views of the NMJs seen in the left panel (A). White arrowheads indicate the loss of nAChR fragments. The progression of the typical degenerative pathology at the NMJ is illustrated, including extensively fragmented nAChR clusters (green colour, B, Panels 1 and 2) and severely damaged nAChR clusters (green colour) with lack of motor nerve terminal innervation (red colour, B, Panel 3).

We examined a total of 512 NMJs in gastrocnemius muscles obtained from CLTI patients undergoing below‐knee amputation surgery. Figure 3 presents representative gastrocnemius NMJ morphology from one CLTI patient. In contrast to control subjects (Figure 1), most NMJs in PAD patients exhibited significant structural alterations. These changes were characterised by fragmented nAChR clusters, with only a few dispersed fragments of nAChRs forming each cluster (Figure 3B). Notably, some nAChR clusters had lost the motor nerve terminal innervation. Figure 4 illustrates the progression of the typical degenerative process at the NMJ, displaying extensively fragmented nAChR clusters (Figure 4B, Panels 1 and 2) and severely damaged nAChR clusters characterised by loss of motor nerve terminal innervation and the absence of the majority of nAChR fragments (Figure 4B, Panel 3).

**FIGURE 5:**
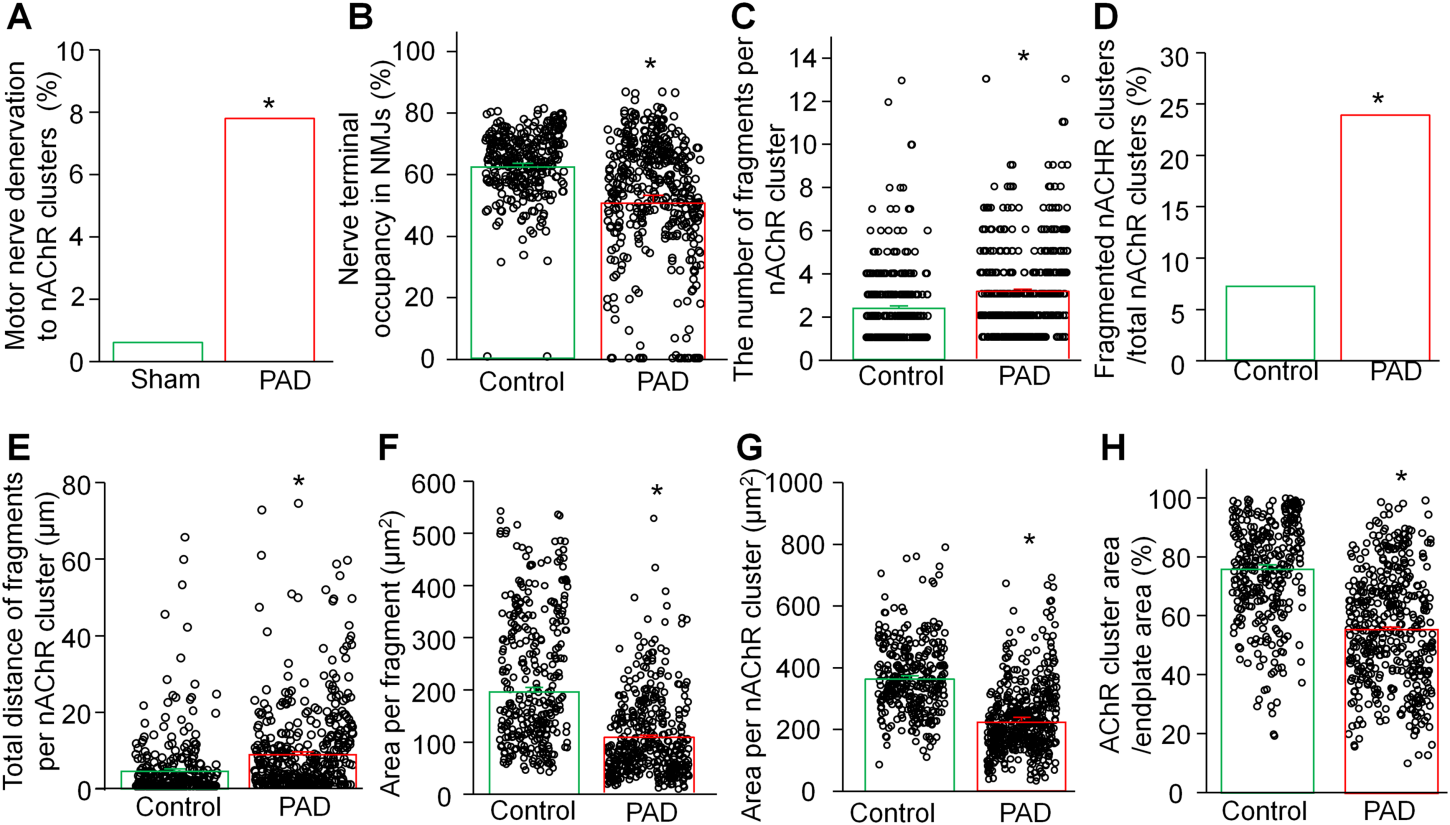
Summary data for several parameters of motor nerve terminals and nAChR clusters in control human subjects and patients with PAD presenting with CLTI. The data are presented as mean ± SEM, with 331 nAChR clusters from three control human subjects and 512 nAChR clusters from 15 PAD patients with CLTI. *p < 0.05 compared with controls.

Quantitative analysis revealed significant differences between PAD patients and control subjects (Figure 5). PAD patients had increased motor nerve denervation to nAChR clusters (7.8% in PAD patients vs. 0.6% in control subjects) and reduced motor nerve terminal occupancy in the NMJs (50.8 ± 2.1% in PAD patients vs. 62.2 ± 1.0 in control subjects, Figure 5A,B). Furthermore, there was severe damage in the nAChR clusters, as shown by an increase in the number of fragments (3.2 ± 0.1 in PAD patients vs. 2.3 ± 0.1 in control subjects), a higher proportion of fragmented nAChR clusters (21.6% in PAD patients vs. 7.6% controls subjects), and an increased total distance between fragments (8.8 ± 0.8 μm in PAD patients vs. 4.6 ± 0.4 μm, Figure 5C–E). Additionally, PAD patients demonstrated a decreased area per fragment (107.1 ± 3.1 μm^2^ in PAD patients vs. 195.9 ± 9.2 μm^2^ in control subjects), a reduced area per nAChR cluster (225.2 ± 5.3 μm^2^ in PAD patients vs. 369.3 ± 6.7 μm^2^), and a diminished nAChR cluster area relative to endplate area (55.7 ± 1.1% in PAD patients vs. 75.7 ± 1.6% in control subjects, Figure 5F–H).

### 3.3 Histological Changes in the Skeletal Muscle of Control Subjects and PAD Patients With CLTI

Histological analysis of muscle samples was conducted for all control subjects and patients with PAD. Specimens of gastrocnemius were fixed in cold methacarn, embedded in paraffin, sectioned at 4 μm and mounted to glass slides. The slide‐mounted specimens were stained with haematoxylin and eosin. Figure S1 presents representative histological images from control and PAD patients. These images reveal appreciable pathology in the calf muscles of PAD patients with CLTI, characterised by concurrent myopathic and neuropathic changes. Myopathic features include internal nuclei, myomyofibre degeneration, round atrophic fibres, and necrotic fibres, while neuropathic alterations manifest as angular fibres and muscle fibre group atrophy. In the more severe cases, there is evidence of significant fibrosis in the muscle specimens (Figure S1D). In advanced stages of PAD, as observed in our patient cohort, it is clear that these neuropathic and myopathic changes intertwine to together contribute to the overall damage of the ischaemic skeletal muscles.

## 4 Discussion

Our study represents the first direct comparison of NMJ morphology between patients with and without arterial occlusive disease providing unequivocal evidence of an association between advanced PAD and pronounced degeneration of NMJ structure within the ischaemic leg muscles. These findings are consistent with and expand upon previous studies demonstrating PAD‐mediated injury to both muscle and nerves in the chronically ischaemic limbs of both patients and animal models of the disease [8, 10, 11, 25]. When patients are evaluated with nerve conduction and detailed electromyographic studies, reductions in nerve function parameters that could be attributed to axonal lesions, demyelination, or denervation‐reinnervation were identified [11, 26, 27, 28, 29]. Furthermore, a relatively common finding in the histological evaluation of PAD muscle is that of muscle fibre group atrophy, which is highly suggestive of focal denervation injury [30, 31, 32], and fibre type grouping indicative of denervation‐reinnervation processes [10, 11, 30, 32, 33, 34]. Work in animal models of critical limb‐threatening ischaemia (the most severe form of PAD, similar to the severity of disease in the patients evaluated in our study) has demonstrated loss of normal NMJ morphology, including motor nerve denervation and fragmentation of nAChR clusters [24, 35].

Our observations share similarities and differences with the findings of a previous study by Jones et al. (2017) [36], who reported more fragmented NMJs in human skeletal muscles across the adult lifespan. Jones et al. used skeletal muscles obtained from surgically discarded materials in 20 patients undergoing lower limb amputation for a variety of clinical indications (including PAD and diabetes). We observed only mild dispersive structures in a few nAChR clusters from the older control subjects (Figure 2) and no dispersive structures in the young trauma donor. The discrepancy between our findings and those of Jones et al. [36] may be attributed to differences in patient populations, clinical indications, or methodological approaches between the two studies. It is important to acknowledge the limitations of using surgically discarded materials as control samples. These tissues may not represent controls without leg pathology, as the legs and their neuromuscular system might have been affected by underlying clinical conditions, such as diabetes and PAD, which are the most common indications for amputation operations. To establish a more accurate baseline for normal human NMJ cellular anatomy, it is essential to obtain sufficient tissue samples from healthy donors with no evidence of PAD, similar to the controls in our study (Figures 1 and 2). While our previous studies in animal models have established a link between deterioration of NMJ morphology (motor nerve denervation and fragmentation of nAChR clusters) and functional abnormalities of the NMJ (decrease in endplate potentials) [21, 22, 24], the extent to which the observed problems in NMJ morphology in PAD patients with CLTI will impact the NMJ function is likely significant but needs further exploration. Future studies should focus on measuring endplate potentials in human samples to address this issue directly and further elucidate the functional consequences of PAD‐induced NMJ degeneration.

In this study, we not only demonstrate significant neuromuscular junction (NMJ) damage in the limbs affected by PAD, but we also document the neuropathic and myopathic changes evident in the histology of the skeletal muscle (Figure S1), which has been reported in previous studies [10, 11, 26, 27, 28, 29, 30, 31, 32]. In the advanced stages of PAD, neuropathic and myopathic processes become increasingly intertwined, making them difficult to distinguish. The pronounced muscle histological changes seen in severe PAD (such as wide variability in myomyofibre size, advanced group atrophy, centrally located nuclei, fibre vacuolisation and target lesions, myomyofibre necrosis with myophagocytosis, thickening of endomysium and perimysium, and fibrofatty infiltration) obscure whether the degeneration arises primarily from myopathy, neuropathy or both, and to what extent each contributes to the overall pathology. We propose that the significant decline in leg function observed in patients with advanced PAD results from the interplay of several factors. Specifically, the ongoing ischaemia due to arterial occlusive disease, combined with damage to neuromuscular junctions and neuropathic and myopathic changes, collectively contribute to the deterioration of function in chronically ischaemic limbs. Our work enhances the understanding of the pathological changes in PAD‐affected nerves and skeletal muscle and provides crucial insights into the mechanisms underlying the severe functional impairments experienced by these patients.

While we employed light microscopy with the labelling of presynaptic nerve terminals and nAChR clusters to evaluate the NMJ structural damage, a limitation of our study is the absence of electron microscopy‐based NMJ pathology evaluation. Electron microscopy can reveal precise ultrastructural abnormalities imperceptible to light microscopy, such as vesicle and mitochondrial distribution near presynaptic membranes, synaptic cleft morphology, and junctional fold integrity in postsynaptic membranes. We plan to incorporate these electron microscopy‐based measurements in future investigations to provide a more comprehensive analysis of the PAD damage to NMJ ultrastructure.

In summary, our study has demonstrated significant damage to the NMJ in skeletal muscles of PAD patients with CLTI. This damage is characterised by motor nerve denervation and fragmentation of the nAChR clusters, as shown by decreased motor innervation, reduced motor nerve terminal occupancy, increased nAChR cluster fragmentation, and diminished nAChR cluster area relative to endplate size. These findings align with and expand upon previous research conducted in both PAD patients and animal models, indicating a correlation between NMJ damage, lower extremity skeletal muscle dysfunction, and poor limb salvage outcomes in the advanced stages of PAD. Our study underscores the potential of targeting the NMJ as a promising strategy in our efforts to enhance skeletal muscle function and address walking impairments and limb salvage challenges in patients with PAD. By exploring NMJ interventions, we may uncover new avenues for improving outcomes in this patient population.

## Author Contributions

All authors designed the experiments. H.T., A.H.H., J.K.K. and Z.Z. performed the experiments, and H.T., A.H.H., Y.T., J.K.K., I.I.P. and Y.L.L. analysed the data and prepared the manuscript. H.T., A.H.H., J.K.K., Z.Z., Y.T., I.I.P. and Y.L.L. wrote, edited, and revised the manuscript. H.T., A.H.H., J.K.K., Z.Z., Y.T., I.I.P. and Y.L.L. approved the version to be published and agreed to be accountable for all aspects of the work.

## Ethics Statement

The study was approved by the Institutional Review Boards at the Nebraska‐Western Iowa Veteran Affairs Health Care System and the University of Nebraska Medical Center.

## Conflicts of Interest

The authors declare no conflicts of interest.

## Supporting information


**Figure S1.** Gastrocnemius muscle histology images from control (A) and CLTI patients (B‐D). Specimens of gastrocnemius were fixed in cold methacarn, embedded in paraffin, sectioned at 4 μm and mounted on glass slides. The slide‐mounted specimens were stained with haematoxylin and eosin. The white bar located in the left lower corner of each slide represents a length of 200 μm.
A‐ control patient with normal muscle histology. The myofibres are polygonal, have similar shape and size and peripherally located nuclei. They are arranged in groups called myofascicles. The myomyofibres are surrounded by a very thin layer of extracellular matrix called endomysium and the myofascicles are surrounded by a very thin layer of extracellular matrix called perimysium.
B‐ CLTI patient with mild neuropathic (angular atrophic fibres, wide distribution of myomyofibre size) and myopathic (internal nuclei, myomyofibre degeneration) pathology.
C‐ CLTI patient with moderate neuropathic (group atrophy of myomyofibres with mild endomysial fibrosis) and myopathic (myomyofibre necrosis, myophagocytosis and mild endomysial fibrosis) pathology.
D‐ CLTI patient with severe neuropathic (group atrophy of myomyofibres with moderate to severe endomysial fibrosis) and myopathic (internal nuclei, myomyofibre degeneration/necrosis and moderate to severe endomysial fibrosis) pathology.

## Data Availability

The data that support the findings of this study are available from the corresponding authors upon reasonable request.
