# Supplementary material for: Neuromuscular Junction Damage in the Calf Muscles of Patients With Advanced Peripheral Artery Disease
Source: Neuropathol Appl Neurobiol. 2025 Feb 24;51(1):e70008. doi: 10.1111/nan.70008 (PMC11848508; doi:10.1111/nan.70008)
Supplement: Supplementary file 1 — Figure S1. Gastrocnemius muscle histology images from control (A) and CLTI patients (B‐D). Specimens of gastrocnemius were fixed in cold methacarn, embedded in paraffin, sectioned at 4 μm and mounted on glass slides. The slide‐mounted specimens were stained with haematoxylin and eosin. The white bar located in the left lower corner of each slide represents a length of 200 μm. A‐ control patient with normal muscle histology. The myofibres are polygonal, have similar shape and size and peripherally located nuclei. They are arranged in groups called myofascicles. The myomyofibres are surrounded by a very thin layer of extracellular matrix called endomysium and the myofascicles are surrounded by a very thin layer of extracellular matrix called perimysium. B‐ CLTI patient with mild neuropathic (angular atrophic fibres, wide distribution of myomyofibre size) and myopathic (internal nuclei, myomyofibre degeneration) pathology. C‐ CLTI patient with moderate neuropathic (group atrophy of myomyofibres with mild endomysial fibrosis) and myopathic (myomyofibre necrosis, myophagocytosis and mild endomysial fibrosis) pathology. D‐ CLTI patient with severe neuropathic (group atrophy of myomyofibres with moderate to severe endomysial fibrosis) and myopathic (internal nuclei, myomyofibre degeneration/necrosis and moderate to severe endomysial fibrosis) pathology. [file NAN-51-e70008-s001.docx]

Figure 1S. Gastrocnemius muscle histology images from control (A) and CLTI patients (B-D). Specimens of gastrocnemius were fixed in cold methacarn, embedded in paraffin, sectioned at 4 μm and mounted on glass slides. The slide-mounted specimens were stained with haematoxylin and eosin. The white bar located in the left lower corner of each slide represents a length of 200 microns.


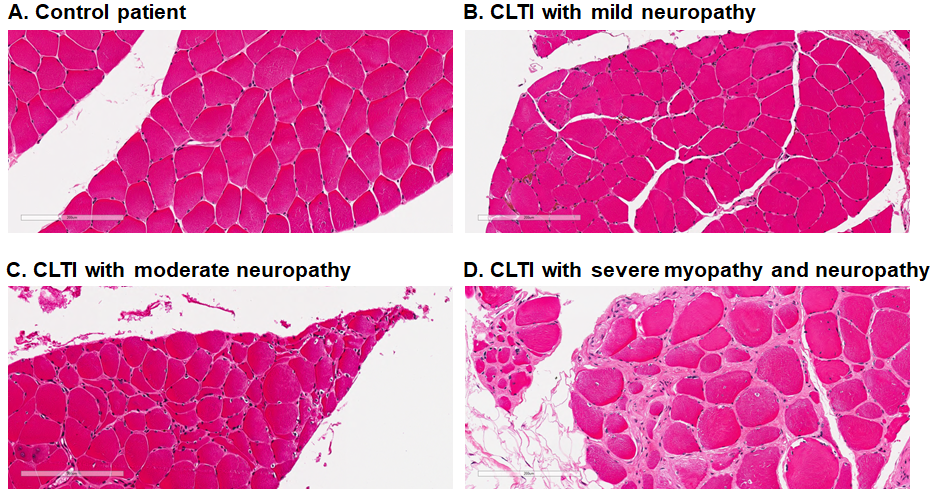


A- control patient with normal muscle histology. The myofibers are polygonal, have similar shape and size and peripherally located nuclei. They are arranged in groups called myofascicles. The myofibers are surrounded by a very thin layer of extracellular matrix called endomysium and the myofascicles are surrounded by a very thin layer of extracellular matrix called perimysium.

B- CLTI patient with mild neuropathic (angular atrophic fibres, wide distribution of myofiber size) and myopathic (internal nuclei, myofiber degeneration) pathology.

C- CLTI patient with moderate neuropathic (group atrophy of myofibers with mild endomysial fibrosis) and myopathic (myofiber necrosis, myophagocytosis and mild endomysial fibrosis) pathology.

D- CLTI patient with severe neuropathic (group atrophy of myofibers with moderate to severe endomysial fibrosis) and myopathic (internal nuclei, myofiber degeneration/necrosis and moderate to severe endomysial fibrosis) pathology.
